# *catena*-Poly[zinc(II)-bis­[μ-5-(2-amino­phenyl)tetra­zolato]-κ^3^ *N* ^1^,*N* ^5^:*N* ^2^;κ^3^ *N* ^2^:*N* ^1^,*N* ^5^]

**DOI:** 10.1107/S1600536808021946

**Published:** 2008-07-16

**Authors:** Xiao-Chun Wen

**Affiliations:** a Ordered Matter Science Research Center, College of Chemistry and Chemical Engineering, Southeast University, Nanjing 210096, People’s Republic of China

## Abstract

The polymeric title compound, [Zn(C_7_H_6_N_5_)_2_]_*n*_, was synthesized by the hydro­thermal reaction of Zn(NO_3_)_2_ with 2-amino­benzonitrile in the presence of NaN_3_. The zinc(II) metal centre displays a distorted octa­hedral coordination environment provided by N atoms of two bidentate chelating and two monodentate 5-(2-amino­phen­yl)tetra­zolate ligands. These ligands act as bridges, linking adjacent Zn atoms into polymeric criss-crossed chains parallel to the [110] and [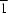
10] directions. Intra­chain N—H⋯N hydrogen-bonding inter­actions are observed.

## Related literature

For the applications of tetra­zole compounds, see: Arp *et al.* (2000 ▶); Dunica *et al.* (1991 ▶); Wang *et al.* (2004 ▶, 2005 ▶); Wittenberger & Donner (1993 ▶).
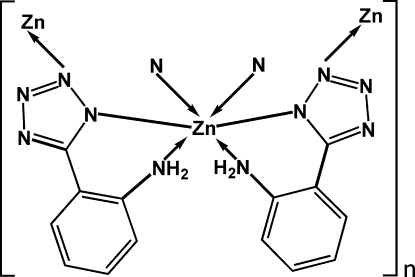


## Experimental

### 

#### Crystal data

[Zn(C_7_H_6_N_5_)_2_]
*M* *_r_* = 385.71
Monoclinic, 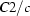

*a* = 10.6751 (16) Å
*b* = 10.9051 (14) Å
*c* = 25.321 (5) Å
β = 94.972 (13)°
*V* = 2936.6 (8) Å^3^
*Z* = 8
Mo *K*α radiation
μ = 1.70 mm^−1^
*T* = 298 (2) K
0.28 × 0.12 × 0.10 mm

#### Data collection

Rigaku Mercury2 diffractometer
Absorption correction: multi-scan (*CrystalClear*; Rigaku, 2005 ▶) *T*_min_ = 0.783, *T*_max_ = 0.844
14618 measured reflections
3343 independent reflections
2859 reflections with *I* > 2σ(*I*)
*R*_int_ = 0.044

#### Refinement

*R*[*F* ^2^ > 2σ(*F* ^2^)] = 0.038
*wR*(*F* ^2^) = 0.092
*S* = 1.09
3343 reflections
226 parameters
H-atom parameters constrained
Δρ_max_ = 0.37 e Å^−3^
Δρ_min_ = −0.70 e Å^−3^

### 

Data collection: *CrystalClear* (Rigaku, 2005 ▶); cell refinement: *CrystalClear*; data reduction: *CrystalClear*; program(s) used to solve structure: *SHELXS97* (Sheldrick, 2008 ▶); program(s) used to refine structure: *SHELXL97* (Sheldrick, 2008 ▶); molecular graphics: *SHELXTL/PC* (Sheldrick, 2008 ▶); software used to prepare material for publication: *SHELXTL/PC*.

## Supplementary Material

Crystal structure: contains datablocks I, global. DOI: 10.1107/S1600536808021946/rz2234sup1.cif

Structure factors: contains datablocks I. DOI: 10.1107/S1600536808021946/rz2234Isup2.hkl

Additional supplementary materials:  crystallographic information; 3D view; checkCIF report

## Figures and Tables

**Table 1:** Hydrogen-bond geometry (Å, °)

*D*—H⋯*A*	*D*—H	H⋯*A*	*D*⋯*A*	*D*—H⋯*A*
N10—H10*A*⋯N6^i^	0.90	2.26	3.095 (3)	154
N9—H9*A*⋯N2^ii^	0.90	2.27	3.108 (3)	155
N9—H9*B*⋯N1^iii^	0.90	2.38	3.246 (3)	160
N9—H9*B*⋯N2^iii^	0.90	2.57	3.242 (3)	132

Symmetry codes: (i) 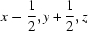; (ii) 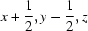; (iii) 
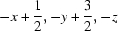
.

**Fig. 1.**
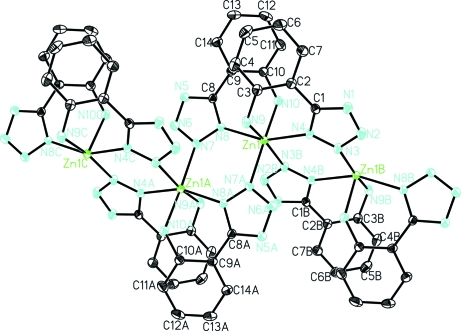
View of the title compound with the atomic numbering scheme. Displacement ellipsoids are drawn at the 30% probability level. H atoms have been omitted for clarity. [Symmetry codes: (A) 1/2-x, 1/2-y,-z; (B) -x, 1-y, -z; (C) 1/2+x, y-1/2, z].

**Fig. 2.**
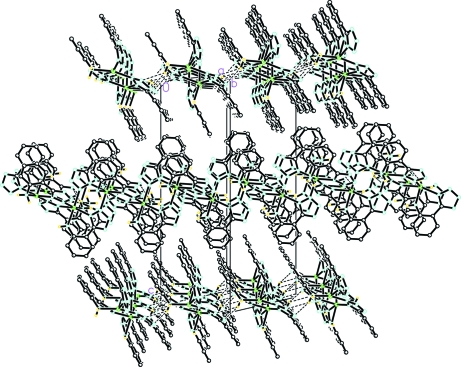
Crystal packing of the title compound viewed along the viewed along the [-110] direction. Intrachain hydrogen bonds are shwn as dashed lines. Hydrogen atoms not involved in hydrogen bonding are omitted for clarity.

## supplementary crystallographic information

### Comment

The tetrazole functional group has found a wide range of applications in coordination chemistry as polydentate ligand, in medicinal chemistry as a metabolically stable surrogate of the carboxylic group, and in materials science as the field of high density energy materials (Wang *et al.*, 2005; Dunica *et al.*, 1991; Wittenberger & Donner, 1993). The crystal structure of the polymeric title compound is reported here.

The compound is isostructural with the corresponding cadmium(II) derivative (Wang *et al.*, 2004). The asymmetric unit of the title compound consists of one zinc(II) atom and two 5-(2-aminobenzyl)tetrazolato ligands. The benzene and tetrazole rings are twisted from each other at dihedral angles of 27.39(14 ) and 21.59(14 )°. Bond distances and angles within the tetrazole rings fall in the usual ranges (Wang *et al.*, 2005; Arp *et al.*, 2000). The metal centre exhibits a distorted octahedral coordination environment (Fig. 1) provided by N atoms of two bidentate-chelating and two monodentate ligands. Both independent ligands act as bridges linking adjacent zinc(II) atoms into polymeric criss-crossed chains parallel to the [110] and [-110] directions (Fig. 2). Intrachain N—H···N hydrogen bonding interactions (Table 1) are present.

### Experimental

A mixture of 2-aminobenzonitrile (0.2 mmol), NaN_3_ (0.4 mmol), Zn(NO_3_)_2_(0.15 mmol) ethanol (1 ml) and a few drops of water was sealed in a glass tube and maintained at 120 °C. Colourless block crystals suitable for X-ray analysis were obtained after 3 days.

### Refinement

All H atoms attached to C and N atoms were fixed geometrically and treated as riding with C—H = 0.93 Å and U_iso_(H) = 1.2U_eq_(C), N—H = 0.90 Å and U_iso_(H) = 1.5U_eq_(N).

### Crystal data

**Table:**

[Zn(C_7_H_6_N_5_)_2_]	*F*_000_ = 1568
*M**_r_* = 385.71	*D*_x_ = 1.745 Mg m^−^^3^
Monoclinic, *C*2/*c*	Mo *K*α radiation λ = 0.71073 Å
Hall symbol: -C 2yc	Cell parameters from 3668 reflections
*a* = 10.6751 (16) Å	θ = 3.0–27.5º
*b* = 10.9051 (14) Å	µ = 1.70 mm^−^^1^
*c* = 25.321 (5) Å	*T* = 298 (2) K
β = 94.972 (13)º	Block, colourless
*V* = 2936.6 (8) Å^3^	0.28 × 0.12 × 0.10 mm
*Z* = 8	

### Data collection

**Table:**

Rigaku Mercury2 diffractometer	3343 independent reflections
Radiation source: fine-focus sealed tube	2859 reflections with *I* > 2σ(*I*)
Monochromator: graphite	*R*_int_ = 0.044
Detector resolution: 13.6612 pixels mm^-1^	θ_max_ = 27.5º
*T* = 298(2) K	θ_min_ = 3.0º
ω scans	*h* = −13→13
Absorption correction: multi-scan(CrystalClear; Rigaku, 2005)	*k* = −14→14
*T*_min_ = 0.783, *T*_max_ = 0.844	*l* = −32→32
14618 measured reflections	

### Refinement

**Table:**

Refinement on *F*^2^	Secondary atom site location: difference Fourier map
Least-squares matrix: full	Hydrogen site location: inferred from neighbouring sites
*R*[*F*^2^ > 2σ(*F*^2^)] = 0.038	H-atom parameters constrained
*wR*(*F*^2^) = 0.092	*w* = 1/[σ^2^(*F*_o_^2^) + (0.0386*P*)^2^ + 3.3164*P*] where *P* = (*F*_o_^2^ + 2*F*_c_^2^)/3
*S* = 1.09	(Δ/σ)_max_ = 0.001
3343 reflections	Δρ_max_ = 0.37 e Å^−^^3^
226 parameters	Δρ_min_ = −0.70 e Å^−^^3^
Primary atom site location: structure-invariant direct methods	Extinction correction: none

### Special details

**Table:**

Geometry. All esds (except the esd in the dihedral angle between two l.s. planes) are estimated using the full covariance matrix. The cell esds are taken into account individually in the estimation of esds in distances, angles and torsion angles; correlations between esds in cell parameters are only used when they are defined by crystal symmetry. An approximate (isotropic) treatment of cell esds is used for estimating esds involving l.s. planes.
Refinement. Refinement of F^2^ against ALL reflections. The weighted R-factor wR and goodness of fit S are based on F^2^, conventional R-factors R are based on F, with F set to zero for negative F^2^. The threshold expression of F^2^ > 2sigma(F^2^) is used only for calculating R-factors(gt) etc. and is not relevant to the choice of reflections for refinement. R-factors based on F^2^ are statistically about twice as large as those based on F, and R- factors based on ALL data will be even larger.

### Fractional atomic coordinates and isotropic or equivalent isotropic displacement parameters (Å^2^)

**Table:**

	*x*	*y*	*z*	*U*_iso_*/*U*_eq_	
Zn1	0.16630 (3)	0.40663 (2)	0.032125 (11)	0.02512 (10)	
N4	0.11712 (19)	0.60243 (17)	0.02478 (8)	0.0249 (4)	
N10	0.0847 (2)	0.4084 (2)	0.10837 (9)	0.0323 (5)	
H10A	0.0285	0.4696	0.1097	0.048*	
H10B	0.0438	0.3365	0.1038	0.048*	
C10	0.1638 (2)	0.4052 (2)	0.15692 (10)	0.0288 (5)	
N9	0.3503 (2)	0.49221 (19)	0.04291 (9)	0.0310 (5)	
H9A	0.4105	0.4343	0.0445	0.046*	
H9B	0.3521	0.5348	0.0125	0.046*	
C9	0.2582 (2)	0.3162 (2)	0.16320 (9)	0.0272 (5)	
N8	0.24495 (18)	0.23775 (18)	0.06944 (8)	0.0245 (4)	
C1	0.1781 (2)	0.6906 (2)	0.05410 (10)	0.0252 (5)	
C2	0.2884 (2)	0.6711 (2)	0.09210 (10)	0.0283 (5)	
C14	0.3353 (3)	0.3134 (3)	0.21067 (10)	0.0346 (6)	
H14A	0.3983	0.2545	0.2154	0.041*	
C3	0.3720 (2)	0.5736 (2)	0.08704 (11)	0.0291 (5)	
C13	0.3197 (3)	0.3964 (3)	0.25076 (12)	0.0438 (7)	
H13A	0.3718	0.3934	0.2821	0.053*	
C4	0.4704 (3)	0.5554 (3)	0.12574 (13)	0.0404 (7)	
H4A	0.5265	0.4913	0.1222	0.048*	
C7	0.3079 (3)	0.7491 (3)	0.13564 (11)	0.0360 (6)	
H7A	0.2553	0.8163	0.1385	0.043*	
C6	0.4044 (3)	0.7281 (3)	0.17470 (12)	0.0437 (7)	
H6A	0.4142	0.7788	0.2043	0.052*	
C5	0.4859 (3)	0.6316 (3)	0.16940 (13)	0.0451 (7)	
H5A	0.5515	0.6177	0.1953	0.054*	
C11	0.1483 (3)	0.4882 (3)	0.19728 (12)	0.0414 (7)	
H11A	0.0852	0.5470	0.1931	0.050*	
C12	0.2265 (3)	0.4838 (3)	0.24397 (12)	0.0473 (8)	
H12A	0.2160	0.5402	0.2708	0.057*	
C8	0.2794 (2)	0.2265 (2)	0.12170 (9)	0.0250 (5)	
N6	0.3457 (2)	0.0666 (2)	0.08495 (9)	0.0380 (6)	
N5	0.3411 (2)	0.1218 (2)	0.13199 (9)	0.0368 (5)	
N7	0.2890 (2)	0.13536 (19)	0.04763 (8)	0.0280 (4)	
N1	0.1206 (2)	0.79890 (19)	0.04653 (9)	0.0330 (5)	
N2	0.0204 (2)	0.77701 (19)	0.01204 (9)	0.0334 (5)	
N3	0.01805 (19)	0.65985 (19)	−0.00074 (9)	0.0282 (5)	

### Atomic displacement parameters (Å^2^)

**Table:**

	*U*^11^	*U*^22^	*U*^33^	*U*^12^	*U*^13^	*U*^23^
Zn1	0.02992 (17)	0.02214 (16)	0.02306 (16)	0.00230 (11)	0.00100 (11)	0.00016 (11)
N4	0.0276 (10)	0.0202 (10)	0.0265 (10)	0.0028 (8)	0.0004 (8)	0.0011 (8)
N10	0.0334 (12)	0.0362 (12)	0.0277 (11)	0.0113 (9)	0.0041 (9)	0.0050 (9)
C10	0.0350 (14)	0.0262 (12)	0.0257 (12)	0.0023 (10)	0.0051 (10)	0.0031 (10)
N9	0.0311 (11)	0.0215 (10)	0.0408 (13)	0.0045 (9)	0.0056 (9)	0.0009 (9)
C9	0.0312 (13)	0.0276 (12)	0.0230 (12)	−0.0015 (10)	0.0028 (10)	0.0027 (10)
N8	0.0295 (10)	0.0217 (10)	0.0221 (10)	0.0045 (8)	0.0017 (8)	0.0006 (8)
C1	0.0270 (12)	0.0208 (11)	0.0284 (12)	0.0000 (9)	0.0056 (9)	0.0001 (10)
C2	0.0278 (12)	0.0253 (12)	0.0317 (13)	−0.0025 (10)	0.0016 (10)	0.0010 (10)
C14	0.0381 (15)	0.0354 (14)	0.0292 (13)	−0.0008 (12)	−0.0025 (11)	0.0022 (11)
C3	0.0292 (13)	0.0240 (12)	0.0343 (14)	−0.0020 (10)	0.0029 (10)	0.0024 (10)
C13	0.0549 (19)	0.0487 (18)	0.0266 (14)	−0.0089 (15)	−0.0029 (13)	−0.0062 (13)
C4	0.0330 (14)	0.0339 (14)	0.0528 (18)	0.0008 (12)	−0.0044 (13)	0.0080 (13)
C7	0.0381 (15)	0.0323 (14)	0.0371 (15)	−0.0016 (12)	0.0011 (12)	−0.0038 (12)
C6	0.0459 (17)	0.0455 (17)	0.0382 (16)	−0.0079 (14)	−0.0051 (13)	−0.0047 (14)
C5	0.0386 (16)	0.0472 (17)	0.0467 (18)	−0.0071 (14)	−0.0130 (13)	0.0094 (14)
C11	0.0539 (18)	0.0321 (14)	0.0396 (16)	0.0081 (13)	0.0128 (13)	−0.0022 (12)
C12	0.068 (2)	0.0427 (17)	0.0322 (15)	−0.0033 (16)	0.0106 (14)	−0.0131 (13)
C8	0.0256 (12)	0.0245 (12)	0.0248 (12)	0.0030 (10)	0.0019 (9)	0.0042 (10)
N6	0.0532 (15)	0.0346 (12)	0.0270 (12)	0.0154 (11)	0.0081 (10)	0.0042 (10)
N5	0.0485 (14)	0.0376 (12)	0.0245 (11)	0.0164 (11)	0.0034 (10)	0.0034 (10)
N7	0.0353 (11)	0.0245 (10)	0.0248 (11)	0.0062 (9)	0.0063 (9)	0.0007 (8)
N1	0.0308 (11)	0.0243 (11)	0.0426 (13)	0.0009 (9)	−0.0042 (10)	−0.0014 (10)
N2	0.0309 (11)	0.0233 (11)	0.0453 (14)	0.0024 (9)	−0.0012 (10)	0.0013 (10)
N3	0.0283 (11)	0.0243 (11)	0.0318 (11)	0.0027 (8)	0.0006 (9)	0.0029 (9)

### Geometric parameters (Å, °)

**Table:**

Zn1—N7^i^	2.164 (2)	C2—C3	1.401 (4)
Zn1—N9	2.170 (2)	C14—C13	1.381 (4)
Zn1—N3^ii^	2.181 (2)	C14—H14A	0.9300
Zn1—N10	2.186 (2)	C3—C4	1.387 (4)
Zn1—N8	2.2027 (19)	C13—C12	1.377 (4)
Zn1—N4	2.2028 (19)	C13—H13A	0.9300
N4—N3	1.345 (3)	C4—C5	1.381 (5)
N4—C1	1.348 (3)	C4—H4A	0.9300
N10—C10	1.430 (3)	C7—C6	1.384 (4)
N10—H10A	0.9000	C7—H7A	0.9300
N10—H10B	0.9001	C6—C5	1.380 (4)
C10—C11	1.386 (4)	C6—H6A	0.9300
C10—C9	1.397 (3)	C5—H5A	0.9300
N9—C3	1.430 (3)	C11—C12	1.388 (4)
N9—H9A	0.9000	C11—H11A	0.9300
N9—H9B	0.9001	C12—H12A	0.9300
C9—C14	1.398 (3)	C8—N5	1.333 (3)
C9—C8	1.467 (3)	N6—N7	1.312 (3)
N8—C8	1.348 (3)	N6—N5	1.339 (3)
N8—N7	1.348 (3)	N7—Zn1^i^	2.164 (2)
C1—N1	1.337 (3)	N1—N2	1.342 (3)
C1—C2	1.470 (3)	N2—N3	1.318 (3)
C2—C7	1.394 (4)	N3—Zn1^ii^	2.181 (2)
			
N7^i^—Zn1—N9	86.46 (8)	C7—C2—C1	119.1 (2)
N7^i^—Zn1—N3^ii^	81.63 (8)	C3—C2—C1	122.0 (2)
N9—Zn1—N3^ii^	165.18 (8)	C13—C14—C9	121.2 (3)
N7^i^—Zn1—N10	164.38 (9)	C13—C14—H14A	119.4
N9—Zn1—N10	108.10 (9)	C9—C14—H14A	119.4
N3^ii^—Zn1—N10	84.76 (9)	C4—C3—C2	119.5 (3)
N7^i^—Zn1—N8	96.71 (7)	C4—C3—N9	121.7 (2)
N9—Zn1—N8	89.91 (8)	C2—C3—N9	118.8 (2)
N3^ii^—Zn1—N8	100.18 (8)	C12—C13—C14	119.6 (3)
N10—Zn1—N8	78.16 (8)	C12—C13—H13A	120.2
N7^i^—Zn1—N4	101.33 (8)	C14—C13—H13A	120.2
N9—Zn1—N4	78.48 (8)	C5—C4—C3	120.7 (3)
N3^ii^—Zn1—N4	95.19 (8)	C5—C4—H4A	119.6
N10—Zn1—N4	87.46 (8)	C3—C4—H4A	119.6
N8—Zn1—N4	157.80 (8)	C6—C7—C2	121.0 (3)
N3—N4—C1	104.77 (18)	C6—C7—H7A	119.5
N3—N4—Zn1	131.56 (16)	C2—C7—H7A	119.5
C1—N4—Zn1	123.01 (16)	C5—C6—C7	119.6 (3)
C10—N10—Zn1	120.53 (17)	C5—C6—H6A	120.2
C10—N10—H10A	109.5	C7—C6—H6A	120.2
Zn1—N10—H10A	110.9	C6—C5—C4	120.2 (3)
C10—N10—H10B	109.5	C6—C5—H5A	119.9
Zn1—N10—H10B	96.0	C4—C5—H5A	119.9
H10A—N10—H10B	109.5	C10—C11—C12	120.3 (3)
C11—C10—C9	120.0 (2)	C10—C11—H11A	119.9
C11—C10—N10	121.2 (2)	C12—C11—H11A	119.9
C9—C10—N10	118.8 (2)	C13—C12—C11	120.3 (3)
C3—N9—Zn1	116.66 (16)	C13—C12—H12A	119.8
C3—N9—H9A	109.5	C11—C12—H12A	119.8
Zn1—N9—H9A	109.8	N5—C8—N8	111.0 (2)
C3—N9—H9B	109.5	N5—C8—C9	122.4 (2)
Zn1—N9—H9B	101.6	N8—C8—C9	126.6 (2)
H9A—N9—H9B	109.5	N7—N6—N5	109.5 (2)
C10—C9—C14	118.6 (2)	C8—N5—N6	105.5 (2)
C10—C9—C8	122.4 (2)	N6—N7—N8	109.5 (2)
C14—C9—C8	119.0 (2)	N6—N7—Zn1^i^	115.23 (16)
C8—N8—N7	104.58 (19)	N8—N7—Zn1^i^	132.09 (16)
C8—N8—Zn1	123.94 (16)	C1—N1—N2	105.5 (2)
N7—N8—Zn1	130.57 (15)	N3—N2—N1	109.2 (2)
N1—C1—N4	110.9 (2)	N2—N3—N4	109.6 (2)
N1—C1—C2	123.6 (2)	N2—N3—Zn1^ii^	114.14 (16)
N4—C1—C2	125.4 (2)	N4—N3—Zn1^ii^	131.85 (16)
C7—C2—C3	118.9 (2)		

Symmetry codes: (i) −*x*+1/2, −*y*+1/2, −*z*; (ii) −*x*, −*y*+1, −*z*.

### Hydrogen-bond geometry (Å, °)

**Table:**

*D*—H···*A*	*D*—H	H···*A*	*D*···*A*	*D*—H···*A*
N10—H10A···N6^iii^	0.90	2.26	3.095 (3)	154
N9—H9A···N2^iv^	0.90	2.27	3.108 (3)	155
N9—H9B···N1^v^	0.90	2.38	3.246 (3)	160
N9—H9B···N2^v^	0.90	2.57	3.242 (3)	132

Symmetry codes: (iii) *x*−1/2, *y*+1/2, *z*; (iv) *x*+1/2, *y*−1/2, *z*; (v) −*x*+1/2, −*y*+3/2, −*z*.
